# 3D Mass Spectrometry Imaging Reveals a Very Heterogeneous Drug Distribution in Tumors

**DOI:** 10.1038/srep37027

**Published:** 2016-11-14

**Authors:** S. Giordano, L. Morosi, P. Veglianese, S. A. Licandro, R. Frapolli, M. Zucchetti, G. Cappelletti, L. Falciola, V. Pifferi, S. Visentin, M. D’Incalci, E. Davoli

**Affiliations:** 1 Environmental Health Sciences Department, Mass Spectrometry Laboratory, IRCCS Istituto di Ricerche Farmacologiche Mario Negri, Via La Masa 19, 20156 Milano, Italy; 2 Oncology Department, Cancer Pharmacology Laboratory, IRCCS Istituto di Ricerche Farmacologiche Mario Negri, Via La Masa 19, 20156 Milano, Italy; 3 Neuroscience Department, Biology of Neurodegenerative Disorders Laboratory, IRCCS Istituto di Ricerche Farmacologiche Mario Negri, Via La Masa 19, 20156 Milano, Italy; 4 Chemistry Department, University of Milano, Via Golgi 19, 20133 Milano, Italy; 5 Department of Molecular Biotechnology and Health Science, University of Torino, Via Nizza 52, 10126 Torino, Italy

## Abstract

Mass Spectrometry Imaging (MSI) is a widespread technique used to qualitatively describe in two dimensions the distribution of endogenous or exogenous compounds within tissue sections. Absolute quantification of drugs using MSI is a recent challenge that just in the last years has started to be addressed. Starting from a two dimensional MSI protocol, we developed a three-dimensional pipeline to study drug penetration in tumors and to develop a new drug quantification method by MALDI MSI. Paclitaxel distribution and concentration in different tumors were measured in a 3D model of Malignant Pleural Mesothelioma (MPM), which is known to be a very heterogeneous neoplasm, highly resistant to different drugs. The 3D computational reconstruction allows an accurate description of tumor PTX penetration, adding information about the heterogeneity of tumor drug distribution due to the complex microenvironment. The use of an internal standard, homogenously sprayed on tissue slices, ensures quantitative results that are similar to those obtained using HPLC. The 3D model gives important information about the drug concentration in different tumor sub-volumes and shows that the great part of each tumor is not reached by the drug, suggesting the concept of pseudo-resistance as a further explanation for ineffective therapies and tumors relapse.

Tumor drug resistance has been ascribed for years to a number of mechanisms that influence the uptake, metabolism or proteins and drugs export from cells or change in the drug target^1^,^2^. One of the most important issues in tumors treatment is the limited ability of chemotherapeutic drugs to effectively penetrate tissues and reach all cancer cells at high enough concentrations to inhibit tumor growth. The ability of a molecule to penetrate neoplastic tissue depends on its own physicochemical properties but also on the tumor vascularization and interstitial pressure which depend not only on cancer cell growth but also on the tumor microenvironment^3^,^4^. This microenvironment has a poorly organized vasculature that permits the formation of hypoxic areas with increased interstitial fluid pressure, so they are hard to reach for chemotherapeutic drugs^5^,^6^.

Besides the hemodynamic implications of the tumor vessel network, the extracellular matrix also can affect drug delivery, preventing or slowing penetration of therapeutic molecules^7^. Whatever the mechanism of action of a drug, however, its effectiveness may be reduced because of limited diffusion inside the tumor^8^.

Malignant pleural mesothelioma (MPM) is a very heterogeneous neoplasm, highly resistant to different drugs^9^. The mechanisms of resistance are not fully understood, but recent observations in our laboratory, indicated that mesothelioma cells growing *in vitro* are sensitive to anticancer drugs such as cisplatin or gemcitabine^10^. This led us to speculate that the *in vivo* resistance may be largely related to the inflammatory tumor microenvironment, possibly associated with the low and heterogeneous drug distribution.

Considering intratumor heterogeneity, it is clear that classical pharmacokinetic studies based on HPLC and LC-MS/MS methods on tissue homogenates, lack of information about the spatial distribution of a chemotherapeutic drug inside solid tumors^11^. New methods are therefore needed to thoroughly investigate how a drug localizes inside a tumor, with the aim of developing more effective therapeutic strategies.

All imaging techniques routinely used in clinical practice (MRI, WBA, PET, Infrared Spectroscopy or Fluorescence Microscopy) are useful to display organ anatomy but they lack specificity or sensitivity. Mass spectrometry imaging (MSI) is one of the latest, rapidly growing surface analysis techniques that permits the simultaneous visualization of multiple endogenous or exogenous compounds over a wide mass range, such as peptides and lipids but also small molecules like metabolites or drugs, in cells and tissues^12^,^13^. It provides information about the distribution of a parent compound and its metabolites with high sensitivity and specificity, without the need to label the analyte.

Among all MSI methods Matrix Assisted Laser Desorption Ionization (MALDI) is the most widely used. In a typical MALDI-MSI experiment thin tissue sections are cut and mounted on a steel plate and homogeneously covered with a matrix solution that is needed to absorb the laser energy and promote the desorption/ionization of the analyte molecule in the ion source^14^,^15^.

The most common matrices used in MALDI experiments are generally organic compounds like sinapinic acid (SA), 2,5-dihydroxybenzoic acid (DHB) or a-cyano-4-hydroxycinnamic acid (CHCA) that lead to an excessive fragmentation of the matrix-derived ions resulting in an high background noise in the low mass region, masking the analytes ion signals. Moreover, conventional MALDI matrices co-crystallize with the analytes, affecting the spatial resolution of images obtained when crystals are too large. Beside conventional MALDI matrix, the use of nanoparticles (NPs) as matrices has made molecular imaging by mass spectrometry possible also for small molecules studies thanks to the almost complete absence of background signals from matrix degradation. Another positive effect is that the spatial resolution depends only by instrumental specification like laser spot diameter and not by the crystallization of the matrix^16^. Metallic NPs have been largely employed in MALDI experiments^17^,^18^,^19^,^20^,^21^ and in particular, TiO2 nanoparticles have been used for distribution studies of small endogenous molecules^22^,^23^.

Each tissue section is analyzed by rastering the laser beam across the tissue surface and acquiring a mass spectrum for each discrete spatial point (pixel). A software is used to work through the thousands of mass spectra and create a molecular image in which each pixel represents a selected ion from the mass spectrum^24^. We recently described a MALDI MSI protocol for the two-dimensional analysis of the intratumoral distribution of paclitaxel (PTX), routinely used in clinical practice for the treatment of ovarian and breast cancer^25^.

During the past decade it has been seen that, like for other 3D imaging techniques, serial computational merging of two dimensional mass spectrometric images makes possible to assemble a three-dimensional model of a whole organ, giving a more accurate description of the molecule distribution^26^,^27^. MSI can generate 3D images of different compounds like proteins, peptides, lipids and endogenous or exogenous metabolites^28^.

This study illustrates the potential of 3D MALDI MSI in the field of pharmacology to investigate drug penetration topography in solid tissues. We extended our two-dimensional MSI protocol to analyze the PTX distribution inside tumors in three dimensions.

## Results and Discussion

Mass Spectrometry Imaging is a well established technique that, since its birth in the late 1970s^29^, quickly grew and found application both in clinical^30^,^31^ and pre-clinical^32^ practice. Although the two-dimensional localization of endogenous or exogenous compounds within tissue sections gives important information, the description of the original organ morphology is lost.

Conversely, the three-dimensional overview can give important clues about the mechanisms involved in a pathology. For this reason, the last frontier of MSI is the development of 3D model to deeper investigate the localization of endogenous compounds such as lipids^33^,^34^ or proteins and peptides^26^,^35^.

As the 3D visualization of how a chemotherapeutic drug distributes inside a tumor can lead to a much more intuitive model it can be important also for the development of new effective pharmacological therapies. Moreover, the 3D reconstruction can be helpful for the drug quantification in different tumor sub-volumes.

For this purpose, we developed a MSI protocol for the 3D visualization of the chemotherapeutic drug paclitaxel inside a human Malignant Pleural Mesothelioma (MPM) model, which is known to be a very heterogeneous neoplasm^9^ and consequently difficult to treat.

**Figure 1:**
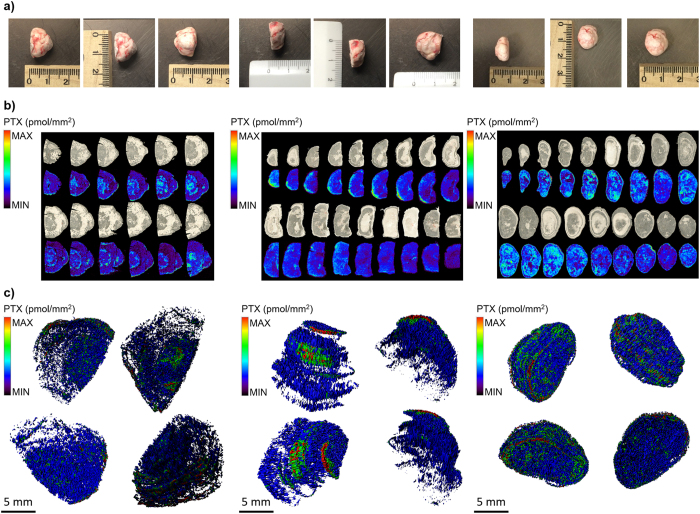
Three-dimensional reconstruction of tumors. (**a**) Mesotheliomas and their dimensions. (**b**) Serial MALDI 2D images of different tumors, compared to optical images of the same slices before acquisition. In each image, colors are normalized to the same scale that appears in different experiments and indicate the same drug concentration. (**c**) 3D representation of PTX distribution inside MPM487 mesotheliomas.

As expected, the 3D-MALDI MSI reconstruction (Fig. 1c), assessed by computational alignment of serial 2D images (Fig. 1b), demonstrates a very heterogeneous distribution of PTX inside the tumors. The videos (Supplementary Movies 1–3) and Figs 1c and 3c, referring to the five-color intensity ranges (Table 1), show how the drug preferably localizes in the edge of the tumors, rather than to the core. The portion colored in red, representing the highest drug concentration, is limited to the peripheral volumes of mesothelioma while in a large part of the model the signal intensity was close or below our limit of detection (uncolored), indicating that the diffusion of PTX inside mesothelioma is seriously hampered by the complexity of tumor structure.

**Figure 2:**
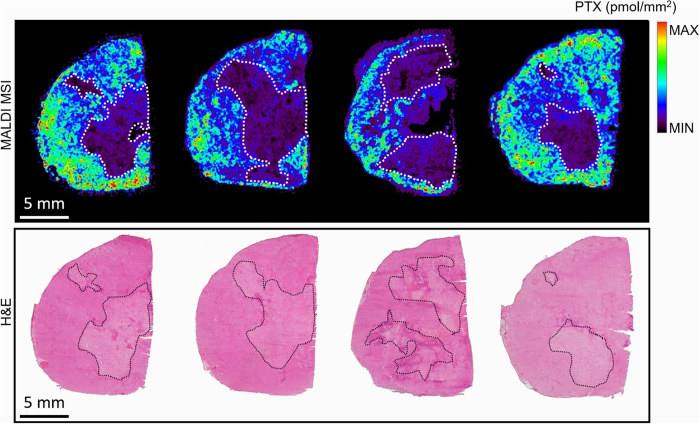
Comparison of PTX distribution by MALDI MSI (upper panel) and H&E staining of the adjacent slice (lower panel). Necrotic areas, highlighted with dashed lines, are those were there is the lower drug signal.

In Fig. 2, it can be observed that the tumor distribution of paclitaxel was associated with proliferating non-necrotic areas, while the lower drug signal in MSI images corresponds to fibrotic and necrotic regions of the tumor section. Comparing MALDI MSI results with the H&E staining of the adjacent slice, it results that drug distribution profile matches with the localization of vital and necrotic areas of the tumor. In these areas the drug is almost absent consistently with what we have shown in ovarian and colon cancer models^13^, supporting the hypothesis of the tumor pseudo-resistance mechanism. According to this hypothesis, cells that are not reached by adequate PTX concentrations are those that may give rise to tumor resistance and relapse^36^. The PTX distribution is probably influenced by the tumor microenvironment where the processes involved in drug delivery are altered or non-functional^8^. Generally, the tumor microenvironment shows an abnormal vasculature that cannot support an homogeneous drug distribution. In addition to the altered extracellular matrix and the lower lymphatic drainage, the abnormal vasculature leads to the accumulation of extravasated macromolecules, increasing the interstitial fluid pressure that is a further an obstacle to drug penetration^37^,^38^,^39^.

**Figure 3:**
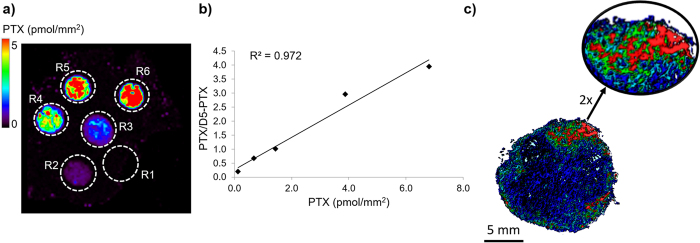
Quantitative analysis by 3D MALDI MSI. (**a**) Control tumor tissue spotted with PTX standards at increasing concentration (R1 = 0 pmol, R2 = 0.5 pmol, R3 = 1 pmol, R4 = 2.5 pmol, R5 = 5 pmol, R6 = 10 pmol). The image shows the intensity of the PTX fragment ion at m/z 284.2 normalized to the D5-PTX fragment ion at m/z 289.2. (**b**) Calibration curve plotted using the mean signal intensity PTX/D5-PTX ratios of the region of interest (ROI) R2-R6. (**c**) 3D images of PTX/D5-PTX distribution inside treated mesothelioma with 2X magnification of the area with the higher signal.

Absolute quantitative analysis by MSI has posed a real challenge and only now are the first publications starting to appear, demonstrating its feasibility^40^. While absolute quantification of PTX was beyond the scope of this work, we estimated PTX concentration in the different tumor volumes, correlating the different PTX ion signal intensity ranges to the concentration values of a calibration curve. The on-tissue quantitative linearity (Fig. 3b) allows reliable measurement of differences in concentration. Semi-quantitative results (Table 1) show that the mean concentration of PTX in mesothelioma was 2.58 μg/g and this was of the same order of magnitude as in the homogenate of the second half of the same tumor measured by HPLC, which was 6.09 μg/g. The highest concentration (in red) was reached only in 0.21% of the tumor volume while a low concentration (<0.2 μg/g, our LOD) was reached in the 37.67% of the tissue. Surprisingly, the drug concentration in certain volumes appeared to be about 500 times higher, than those not reached by the drug (Table 1). This difference in concentration suggests that here the drug may not move by simple diffusion but by mean of other mechanisms that facilitate localization in specific areas (for example where functional vessels are present) while preventing the accumulation in others (higher interstitial fluid pressure, hypoxic, acidic or nutrient-deprived conditions) consistently with alteration of tumor microenvironment^36^.

This pipeline highlights the wide heterogeneity in PTX distribution inside the tumor, giving important clues to the question of tumor drug resistance and relapse. Moreover, it can be applied to identify and to verify the activity of compounds used as enhancers of drug distribution that could increase the therapeutic index of anticancer agents administered in combination such as imatinib^41^ or losartan^42^. The development of a new quantification method based on MALDI imaging could implement the qualitative information with a more informative description of the drug localization. The 3D reconstruction, compared to classical HPLC method, allows an estimation of the drug concentration in different tumor sub-volumes, considering the heterogeneity of tumor microenvironment and identifying areas that are reached by a low drug concentration.

The quantitative 3D rendering reconstruction by MALDI imaging mass spectrometry can bring to a more informative description of the tumor drug distribution, showing large volumes of tumor tissue where drug concentrations are lower than the average, possibly even below the effective dose.

Our findings confirm the important role that the structural heterogeneity of the tumor microenvironment plays in drug distribution, which can even limit the success of therapy. Finally, this observation is of fundamental importance for distinguishing drug-resistance due to cellular resistance mechanisms from pseudo-resistance due to the inadequate drug exposure.

## Methods

### Drugs and reagents

For MSI experiments, paclitaxel (PTX, Indena S.p.A., Milan, Italy) and paclitaxel-D5 (D5-PTX, Toronto Research, Canada) were dissolved in ethanol at a concentration of 1 mg/ml. Serial dilution of PTX were prepared in ethanol 50% from 0.5 to 100 pmol/μL to build the calibration curve used for quantitative analysis. For treatment purpose, PTX was dissolved in 50% Cremophor EL (Sigma) and 50% ethanol and further diluted in saline immediately before use.TiO_2_ nanoparticles (Aeroxide® TiO_2_ P25, Evonik Industrials, Essen, Germany), used as matrix for MSI experiments, were dissolved in 50% ethanol, KCl 0.5% at a concentration of 1 mg/mL. In order to avoid the agglomeration and sedimentation of TiO_2_ nanoparticles, they were vortexed and sonicated for 15 minutes just before use, and added with D5-PTX (3 μg/mL) as internal standard.

### Mice and Human Xenografts

Procedures involving animals and their care were conducted in conformity with the following laws, regulations and policies governing the care and use of laboratory animals: Italian Governing Law (D.lgs 26/2014; Authorization n.19/2008-A issued March 6, 2008 by Ministry of Health); Mario Negri Institutional Regulations and Policies providing internal authorization for persons conducting animal experiments (Quality Management System Certificate – UNI EN ISO 9001:2008 – Reg. N° 8576-A); the NIH Guide for the Care and Use of Laboratory Animals (2011 edition) and EU directives and guidelines (EEC Council Directive 2010/63/UE) and in line with Guidelines for the welfare and use of animals in cancer research {Workman, 2010}. The statement of Compliance (Assurance) with the Public Health Service (PHS) Policy on Human Care and Use of Laboratory Animals has been recently reviewed (9/9/2014) and will expire on September 30, 2019 (Animal Welfare Assurance #A5023-01).

Animals experiments has been reviewed and approved by the IRFMN Animal Care and Use Committee (IACUC) that includes members *“ad hoc”* for ethical issues. Animals were housed in the Institute’s Animal Care facilities which meet international standards. They were regularly checked by a certified veterinarian who is responsible for health monitoring, animal welfare supervision, experimental protocols and procedures revision.

MPM487 is a malignant pleural mesothelioma xenograft with biphasic morphology established in our laboratory from patient derived cells (obtained from the Mesothelioma Biobank-Pathology Dept., SS Antonio e Biagio General Hospital, Alessandria, Italy) and maintained by serial passages in nude mice. Six to seven-week-old female NCr-*Nu/Nu* mice (from Envigo, Udine, Italy) were inoculated subcutaneously with tumor fragments of MPM487. Tumor growth was measured with a digital caliper two times a week and tumor volume (mm^3^) was calculated as (length × width^2^ [mm^2^])/2.

When tumor weight reached approximately 1000 mg, mice were treated with vehicle (CTRL) or with a single dose of PTX intravenously (i.v.) at a dose of 60 mg/kg. Animals were sacrificed 4 hours after treatment under CO_2_ and all efforts were made to minimize suffering. Tumors were explanted and then immediately snap-frozen in liquid nitrogen and stored at −80 °C until further analysis.

Mesothelioma harvested from tumor-bearing mice (Fig. 1a) were divided into two equal parts, one to be imaged and the other for HPLC quantitative analysis after tissue homogenation.

### Quantitative Analysis by HPLC

The total concentration of PTX in tumors was determined by HPLC as previously described^43^. Tissues were previously homogenized in 0.2 M CH_3_COONH_4_ pH 4.5 (1:2 wt/vol) and 0.5 mL of homogenate tissues for each study sample was assayed together with a five points of standard calibration curve prepared in the corresponding control tissues obtained from untreated mice at concentrations ranging from 0.1 to 5 μg/sample. The limit of quantification (LOQ) was 0.08 μg/sample.

### Imaging Mass Spectrometry

The visualization of the paclitaxel distribution in tumors was determined by MSI according to the method we recently published^25^.

Frozen control and treated tumors (Fig. 1a) were cut into 10 μm thick sections using a cryo-microtome (Leica Microsystems, Wetzler, Germany) at −20 °C and mounted on pre-cooled MALDI plates (Opti-TOF 384 Well insert) by standard thaw-mounting techniques. One section every 500 μm was cut from treated tumors.

For the quantitative MALDI analysis a calibration curve was built by spotting PTX at increasing concentration (0.5–10 pmol) on a tumor tissue slice treated with vehicle (Fig. 3a). Plates were then dried in a vacuum drier overnight at room temperature. All samples were sprayed with matrix suspensions using a BD 180 precision double-action trigger airbrush (Fengda®, Zhejiang, China) with a 0.20 mm nozzle diameter, using nitrogen at 0.2 atm.D5-PTX was homogeneously sprayed onto tumor sections together with a TiO_2_ nanoparticle-based matrix and used as Internal Standard (IS). Care was taken to avoid over-spraying the matrix suspension on a single point to avoid the formation of droplets that would wet the surface with possible damage of the tissue structure, analyte diffusion or partial detachment of the slice from the plate.

**Figure 4:**
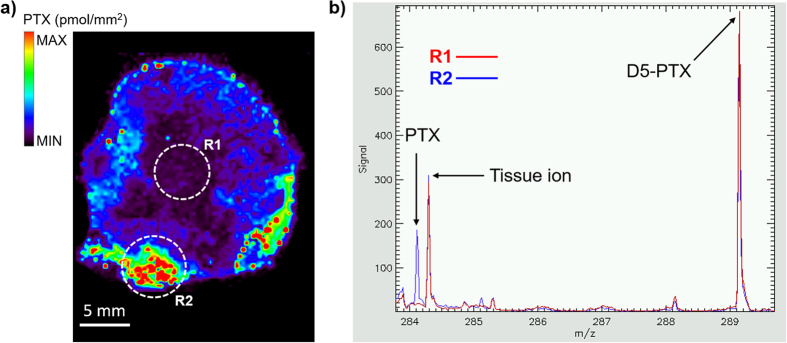
MALDI MSI of paclitaxel distribution inside treated tissue slice. (**a**) Ion image of PTX fragment (m/z 284.2) normalized to the internal standard D5-PTX (m/z 289.2) that was homogenously sprayed all over the tissue sections. Two regions of interest (ROIs, R1 and R2) were drawn in two areas of the tissue with a significant difference of the PTX/D5-PTX signal. (**b**) Mean spectra of R1 and R2. PTX fragment peak intensity at m/z 284.2 significantly decreases in R1 while D5-PTX fragment at m/z 289.2 remains almost constant in R1 e R2.

A MALDI 4800 TOF-TOF (AB SCIEX Old Connecticut Path, Framingham, MA 01701, USA) was used, equipped with a 355 nm Nd:YAG laser with a 200 Hz repetition rate, controlled by the 4000 Series Explorer TM software (AB SCIEX Old Connecticut Path, Framingham, MA 01701, USA). MS spectra were acquired with 20 laser shots with intensity of 6000 arbitrary units, with a bin size of 1.0 ns, acquiring spectra in reflectron negative-ion mode. Images of tissue sections were acquired using the 4800 Imaging Tool Software (www.maldi-msi.org, M. Stoeckli, Novartis Pharma, Basel, Switzerland) with an imaging raster of 100 μm, in order to find a compromise between a good spatial resolution and time requirement for each acquisition. Tissue View software 1.1 (AB SCIEX Old Connecticut Path, Framingham, MA 01701, USA) was used to process and display ions distribution inside the tumor sections, using a classical rainbow color scale. PTX and D5-PTX were imaged by plotting fragment ions at m/z 284.2 and m/z 289.2 respectively, corresponding to side chain with amide-acyl group (Fig. 4b).

Normalization of the PTX ion signal (m/z 284.2) was obtained computationally dividing its intensity by the intensity of the IS (m/z 289.2) pixel by pixel, to compensate ion suppression effects due to different tissue histology^27^.

### 3D Reconstruction and Quantitative Analysis by MSI

For the 3D model reconstruction, all the 2D images were computationally aligned to each other and merged, regaining the initial spatial relations prior to sectioning (Fig. 1c)^44^.

In our experiments, all the 2D images were computationally aligned to each other using Autoaligner® x64 6.0.1 software (Bitplane, Zurich, Switzerland), that uses image features to find the optimal alignment between neighboring slices. Once aligned, 3D models of the tumors were built by merging 2D images using Imaris 8.1 software (Bitplane, Zurich, Switzerland), obtaining volumetric reconstruction of the chemotherapeutic drug distribution.

Quantitative MALDI analysis were done by comparison with a calibration curve (Fig. 3b) built by spotting PTX standards in increasing amounts (0.5–10 pmol) on a mesothelioma tissue slice treated with vehicle (Fig. 3a). From this calibration curve, the maximum PTX concentration was then calculated on the 10 μm-thickness treated tumors slice. The 3D software was used to derive single voxel and overall concentrations in the different hypersurfaces. Concentrations were grouped in five color-coded intensity ranges, used to assign colors for the 3D rendering (Table 1). Black corresponds to the PTX signal intensity in point 0 of the calibration curve (Fig. 3b), standing for the chemical noise of the mass spectrum. The range of concentrations, defined by the 3D software, were related to different tissue weights and volumes. Total drug concentration in the whole tumor was then calculated as a weighted average of PTX concentrations in each hyper-surface.

### Ethics Statements

The IRFMN adheres to the principles set out in the following laws, regulations, and policies governing the care and use of laboratory animals: Italian Governing Law (D.lgs 26/2014; Authorization n.19/2008-A issued March 6, 2008 by Ministry of Health); Mario Negri Institutional Regulations and Policies providing internal authorization for persons conducting animal experiments (Quality Management System Certificate–UNI EN ISO 9001:2008 –Reg. N° 6121); the NIH Guide for the Care and Use of Laboratory Animals (2011 edition) and EU directives and guidelines (EEC Council Directive 2010/63/UE). The Statement of Compliance (Assurance) with the Public Health Service (PHS) Policy on Human Care and Use of Laboratory Animals was recently reviewed (9/9/2014) and will expire on September 30, 2019 (Animal Welfare Assurance #A5023-01). The animals were regularly checked by a certified veterinarian responsible for health monitoring, animal welfare supervision, experimental protocols and procedure revision. All surgery was done under general anesthesia, and all efforts were made to minimize suffering.

## Additional Information

**How to cite this article**: Giordano, S. *et al.* 3D Mass Spectrometry Imaging Reveals a Very Heterogeneous Drug Distribution in Tumors. *Sci. Rep.* **6**, 37027; doi: 10.1038/srep37027 (2016).

**Publisher’s note:** Springer Nature remains neutral with regard to jurisdictional claims in published maps and institutional affiliations.

## Supplementary Material

 Supplementary Video 1

Supplementary Video 2

Supplementary Video 3

Supplementary Information

## Figures and Tables

**Table 1:** Quantitative analysis of paclitaxel inside the MPM487 mesothelioma illustrated in Fig. 3c by 3D MALDI MSI.

Colors assigned to 3D model	PTX range (μg/g)	Tissue weight (mg)	Volume (%)	PTX amount (μg)	PTX tumor conc. (μg/g)
RED	>32.79–≤104.98	1.48	0.21	0.10	2.58
GREEN	>10.24–≤32.79	21.51	3.00	0.46	
LIGHT BLUE	>3.2–≤10.24	113.26	15.77	0.76	
BLUE	>0.2–≤3.2	311.24	43.35	0.53	
BLACK	<LOD	270.50	37.67	0.00	
TOTAL		718.00	100.00	1.86	
