# Supplementary material for: 3D Mass Spectrometry Imaging Reveals a Very Heterogeneous Drug Distribution in Tumors
Source: Sci Rep. 2016 Nov 14;6:37027. doi: 10.1038/srep37027 (PMC5107992; doi:10.1038/srep37027)
Supplement: Supplementary Information [file srep37027-s4.pdf]

3D MASS SPECTROMETRY IMAGING REVEALS A VERY HETEROGENEOUS DRUG DISTRIBUTION IN TUMORS

S. Giordano[1], L. Morosi[2], P. Veglianese[3], S.A. Licandro[2], R. Frapolli[2], M. Zucchetti[2], G. Cappelletti[4], L. Falciola[4], V. Pifferi[4], S. Visentin[5], M. D'Incalci[2] and E. Davoli[1*]

[1]*Environmental Health Sciences Department, Mass Spectrometry Laboratory, IRCCS Istituto di Ricerche Farmacologiche Mario Negri, Via La Masa 19, 20156 Milano, Italy*
[2]*Oncology Department, Cancer Pharmacology Laboratory, IRCCS Istituto di Ricerche Farmacologiche Mario Negri, Via La Masa 19, 20156 Milano, Italy*
[3]*Neuroscience Department, Biology of Neurodegenerative Disorders Laboratory, IRCCS Istituto di Ricerche Farmacologiche Mario Negri, Via La Masa 19, 20156 Milano, Italy*
[4]*Chemistry Department, University of Milano, Via Golgi 19, 20133 Milano, Italy*
[5]*Department of Molecular Biotechnology and Health Science, University of Torino, Via Nizza 52, 10126 Torino, Italy*

*Correspondence to enrico.davoli@marionegri.it

SUPPLEMENTARY

Video 1| Three-dimensional model of PTX distribution inside mesothelioma 1.

Video 2| Three-dimensional model of PTX distribution inside mesothelioma 2.

Video 3| Three-dimensional model of PTX distribution inside mesothelioma 3.
